# Effects of a Mindfulness App on Employee Stress in an Australian Public Sector Workforce: Randomized Controlled Trial

**DOI:** 10.2196/30272

**Published:** 2022-02-10

**Authors:** Larissa Bartlett, Angela J Martin, Michelle Kilpatrick, Petr Otahal, Kristy Sanderson, Amanda L Neil

**Affiliations:** 1 Wicking Dementia Research and Education Centre University of Tasmania Hobart Australia; 2 Menzies Institute for Medical Research University of Tasmania Hobart Australia; 3 School of Health Sciences University of East Anglia Norwich United Kingdom

**Keywords:** mindfulness, stress, apps, smartphone app, employee, workplace, performance, mobile phone

## Abstract

**Background:**

Workplace-based mindfulness programs have good evidence for improving employee stress and mental health outcomes, but less is known about their effects on productivity and citizenship behaviors. Most of the available evidence is derived from studies of mindfulness programs that use class-based approaches. Mindfulness apps can increase access to training, but whether self-directed app use is sufficient to realize benefits equivalent to class-based mindfulness programs is unknown.

**Objective:**

We assessed the effectiveness of a mindfulness app, both with and without supporting classes, for reducing employees’ perceived stress. Changes in mindfulness, mental health, quality of life, perceptions of job demand, control and support, productivity indicators, organizational citizenship, and mindful behaviors at work were also investigated.

**Methods:**

Tasmanian State Service employees were invited by the Tasmanian Training Consortium to a 3-arm randomized controlled trial investigating the effects of a mindfulness app on stress. The app used in the Smiling Mind Workplace Program formed the basis of the intervention. The app includes lessons, activities, and guided meditations, and is supported by 4 instructional emails delivered over 8 weeks. Engagement with the app for 10-20 minutes, 5 days a week, was recommended. Reported data were collected at baseline (time point 0), 3 months from baseline (time point 1 [T1]), and at 6-month follow-up (time point 2). At time point 0, participants could nominate a work-based observer to answer surveys about participants’ behaviors. Eligible participants (n=211) were randomly assigned to self-guided app use plus four 1-hour classes (app+classes: 70/211, 33.2%), self-guided app use (app-only: 71/211, 33.6%), or waitlist control (WLC; 70/211, 33.2%). Linear mixed effects models were used to assess changes in the active groups compared with the WLC at T1 and for a head-to-head comparison of the app+classes and app-only groups at follow-up.

**Results:**

App use time was considerably lower than recommended (app+classes: 120/343 minutes; app-only: 45/343 minutes). Compared with the WLC at T1, no significant change in perceived stress was observed in either active group. However, the app+classes group reported lower psychological distress (β=−1.77, SE 0.75; *P*=.02; Cohen *d*=–0.21) and higher mindfulness (β=.31, SE 0.12; *P*=.01; Cohen *d*=0.19). These effects were retained in the app+classes group at 6 months. No significant changes were observed for the app-only group or for other outcomes. There were no significant changes in observer measures at T1, but by time point 2, the app+classes participants were more noticeably mindful and altruistic at work than app-only participants.

**Conclusions:**

Including classes in the training protocol appears to have motivated engagement and led to benefits, whereas self-guided app use did not realize any significant results. Effect sizes were smaller and less consistent than meta-estimates for class-based mindfulness training.

**Trial Registration:**

Australian New Zealand Clinical Trials Register ACTRN12617001386325; https://www.anzctr.org.au/Trial/Registration/TrialReview.aspx?id=372942&isReview

## Introduction

### Workplace Mindfulness Training

There is growing evidence in support of workplace-based mindfulness programs for increasing employee mindfulness, reducing stress, and improving mental health and well-being [1,2]. In the workplace literature, mindfulness correlates positively with psychological capital, organizational citizenship, and perceived job control and inversely with perceived job demands [3-5]. Accordingly, it is theorized that increasing employee mindfulness through training may help protect against stress, poor mental health, and work-based psychosocial risks. However, few randomized controlled trials (RCTs) have examined the intervention effects of workplace-based mindfulness programs on psychosocial risk factors or organizational outcomes such as employee productivity or performance [1].

Unmanaged stress is known to lead to psychological distress, depression, and anxiety [6,7], which are well-evidenced contributors to lost productivity via higher levels of employee absenteeism and presenteeism [8]. In Australia, the combined annual cost of absenteeism and presenteeism attributable to poor mental health is >US $11 billion, representing a significant economic burden [9]. Furthermore, the consequences of chronic stress include inattentiveness and antisocial or aggressive behavior that can be detrimental to work-based relationships and performance [10].

The occupational health psychology and workplace management literature points to the importance of considering factors that affect employee stress at both the organizational and individual levels [11]. A combined focus on minimizing work-related risk factors for mental health problems, promoting positive aspects of work and fostering employee strengths, and providing tertiary support to address presenting problems is considered best practice [12]. Although redressing adverse working conditions and improving management practices are vital components of workplace well-being strategies, supporting staff to access and develop personal coping strategies is also an important aspect of a healthy work environment [13]. Mindfulness training can provide personal support for employees as it actively cultivates adaptive coping skills that can buffer the effects of stress on employee health and well-being [14,15]. It may also help redress the organizational burden of health-related lost productive time (LPT) by improving mental health [16].

Mindfulness meditation involves the sustained practice of intentionally applying nonjudgmental attention to the current experience. There is some evidence that this practice improves attentional capacities [17], prosocial acting [18], and qualities that influence interpersonal relationships, such as gratitude and forgiveness [19]. Aggression has also been shown to reduce by following mindfulness training [20]. Amassing evidence suggests that increasing mindfulness through training can improve workplace performance, relationships, and well-being [21,22].

### Mindfulness Apps

Smartphone apps are an increasingly popular and accessible mode of delivery for mindfulness training and practice [23]. App functionality enables high-quality multimedia delivery of learning content that can be entirely preprogrammed to maximize intervention integrity and support self-guided learning [24]. For behavioral research, apps also have the ability to record engagement and use data. These data offer a more accurate measure of program engagement than participant recall, which is often used in mindfulness studies [25].

According to a review of 23 mindfulness apps against the Mobile App Rating Scale [26], the top 4 were Headspace, Smiling Mind, iMindfulness, and Mindfulness Daily [23]. The review by Mani [23] noted an absence of RCT evidence for the efficacy of mindfulness apps. Several trials of mindfulness apps have since been published, reporting results for stress, anxiety, depression, and well-being [27-32]. Only one of these RCTs was conducted in a workforce sample [27] in which self-guided use of the Headspace app gave rise to significant small- to moderate-sized effects for well-being, anxiety, depression, and psychosocial risk factors (job control and social support). Thus, this study supports the potential of an app-based workplace-based mindfulness program to positively influence job-related and affect-related variables associated with employee stress [33,34]. However, the effects of app-based workplace-based mindfulness programs have not yet been assessed for changing employee stress appraisals; chronic stress symptomology; or organizational performance outcomes such as productivity, citizenship behaviors, and social interactions [21,22].

### Study Aims

This study examines the efficacy of an app-based, low-dose workplace-based mindfulness program in a large, geographically and occupationally diverse Australian public service workforce. The trial followed an earlier pilot RCT of a 5-week Mindfulness at Work Program within the same workforce [35]. The Mindfulness at Work Program involved five 90-minute in-person classes and prescribed 20 minutes of daily meditation practice. Results of the pilot showed strong effects on stress reduction, mental health, and well-being but no significant improvements in health-related productivity. In-person class attendance at work time was found to be unfeasible for a high proportion of employees due to scheduling and geographical barriers. This study was conceived to examine whether low-dose mindfulness training using a mindfulness app could overcome accessibility challenges and realize beneficial outcomes for employee stress observed in face-to-face programs. The app that underpins the Smiling Mind Workplace Program [36] was selected, as it is already established in the Australian market and ranks highly against the Mobile App Rating Scale criteria [23].

The primary aim of this study is to assess the efficacy of the Smiling Mind Workplace Program app, offered both with and without supporting classes, in reducing employee stress (aim 1). We hypothesize that employees using the Smiling Mind Workplace Program app in conjunction with a series of four 1-hour classes (app+classes group) or using the Smiling Mind Workplace Program app self-guided without supporting classes (app-only group) would each report a consistent moderate-sized reduction in perceived stress when compared with a waitlist control (WLC) group.

The secondary aims are to explore the effects of this low-dose mindfulness intervention on psychological distress, mindfulness, health-related quality of life, perceived job demands, control, and resources (aim 2); explore changes in health-related LPT (aim 3); and explore observer-reported changes in participants’ organizational citizenship and mindful behaviors (aim 4). The effect retention was also investigated (aim 5).

## Methods

### Overview

A 3-arm, open-label, parallel-group RCT was conducted between February 2018 and April 2019. The study was approved by the University of Tasmania health and medical human research ethics committee (H0016587) and registered with the Australian and New Zealand Clinical Trials Register in February 2018 (12617001386325). Baseline data were collected using web-based surveys administered in February 2018 (time point 0 [T0]). Postintervention surveys were conducted 3 months from baseline in May 2018 (time point 1 [T1]), with a 6-month follow-up in July 2018 (time point 2 [T2]). App use data were obtained at T1 and T2. The active intervention groups completed their training between T0 and T1. The control group was invited to access the intervention between T1 and T2. A further data collection wave was conducted 14 months from baseline (time point 3); however, analyses were not conducted because of high (85%) attrition (data not reported).

### Participants

#### Overview

The study sample was drawn from the Tasmanian State Service (TSS). The TSS employs approximately 18,000 people from 18 service agencies and centers across the island state of Tasmania, Australia. TSS employees work in a wide variety of roles (eg, frontline service and professional, administration, information, and asset management and maintenance). An invitation was widely disseminated via email and staff newsletters to express interest in joining a study of app-based mindfulness training for employee stress protection (Multimedia Appendix 1 [37]). The Tasmanian Training Consortium (TTC), which provides TSS staff development and training services, coordinated the invitation dissemination and collated the responses.

Participants needed to have access to a smartphone of any brand for personal use, permission from their supervisor to attend four 1-hour seminars in person or via videoconferencing, and make a commitment to complete the surveys. Eligibility was assessed after baseline based on no concurrent mindfulness or stress-management program of any type, including the use of other mindfulness apps, and not having unmanaged depression or other mental health conditions that might be exacerbated with unsupervised meditation. Mental health eligibility was assessed using baseline survey data from the Patient Health Questionnaire-9 (PHQ-9; [38]) and 2 questions about current and past mental health diagnoses. If respondents indicated a current or previously diagnosed mental health condition or their PHQ-9 score exceeded 15, indicating moderate-to-severe depression symptoms, their study eligibility was subject to review by a registered psychologist.

In the baseline surveys (T0), respondents were asked if they wished to nominate a work-based observer to join the study to answer some questions about the participants’ behaviors at work. If *yes* was selected, the first name and email address of the nominee were entered, and the observer was invited to complete brief surveys about their observations of their paired participant’s behaviors at each of the study time points.

#### Randomization, Blinding, and Consent

An independent statistician (PO) randomized eligible participants into the 3 groups, stratified by whether they had an observer. Group allocations were sent to the TTC, who notified the participants of their training schedule and coordinated the seminars. It was not feasible to blind the TTC staff, study participants, or teacher to treatment [39]. All data were collected via the web using surveys administered using REDCap (Research Electronic Data Capture; Vanderbilt University) [40]. The CHERRIES (Checklist for Reporting Results of Internet e-Surveys) [37] study is included in Multimedia Appendix 1. Research personnel only interacted with randomized participants by email to administer the web-based surveys, and all analyses were conducted on deidentified data. Consent to participate in the research was given at the commencement of each survey, and no incentives were provided. The CONSORT (Consolidated Standards of Reporting Trials) checklist is included in Multimedia Appendix 1.

#### Interventions

Released to the market in 2014, the Smiling Mind Workplace Program aims to enable working adults to develop mindfulness skills and embed mindfulness practices into daily life. The established low-dose mindfulness program involves a series of 5 learning modules delivered in 4 interactive 1-hour face-to-face workshops. These are led by a Smiling Mind facilitator over 8 weeks and supported by the use of the Smiling Mind Workplace Program app. This app comprises 41 elements, including videos and audio lessons, guided meditations, and practical activities such as moving with awareness between meetings, breathing techniques, and listening exercises to help cultivate workplace mindfulness. Use of the app-based activities and meditations is supported by fortnightly emails relating to the content covered in the workshops and app-based lessons. The recommended minimum engagement with the Smiling Mind Workplace Program app is 10 to 20 minutes’ mindfulness practice each weekday. Smiling Mind Workplace Program history and content are provided in Multimedia Appendix 1.

To maximize accessibility, Smiling Mind Workplace Program workshops were delivered in a seminar format in university venues located in the north, northwest, and south of the state. Classes ran twice, in the morning and afternoon, on the advertised dates. Participants were able to attend in person or via videoconferencing, and catch-up recordings were made available. All classes were led by the same mindfulness teacher with certification from the University of Massachusetts Center for Mindfulness and >10 years of teaching experience. No supplementary messaging, incentives, or other forms of contact from the study team were used to encourage intervention engagement.

The app+classes group participants were invited to download and use the Smiling Mind Workplace Program app and attend four 1-hour classes scheduled fortnightly during work time. These participants were sent fortnightly generic emails from the Smiling Mind team to support the use of the app-based materials.

The app-only group participants were invited to download and use the Smiling Mind Workplace Program app and received fortnightly emails but were not invited to attend the classes.

The WLC group participants received no information during T0 to T1. After data collection for T1 was complete, the WLC group was invited to a single 2-hour seminar and to download and use the Smiling Mind Workplace Program app self-guided, in conjunction with the fortnightly emails.

All groups retained access to the Smiling Mind Workplace Program app for 12 months.

### Measures

Demographic variables (age, sex, marital status, educational attainment, work role, and schedule) were collected from participants at T0, as were past or planned exposure to other mindfulness or stress management training and self-ratings of readiness for change (percent).

The 10-item Perceived Stress Scale (PSS; [41]) was used to assess the primary outcome at all time points. Response options were summed (range 0-40), with higher scores indicating higher perceived stress. The baseline PSS data showed good internal consistency (Cronbach α=.92).

The PHQ-9 [38] was used for eligibility screening. Established clinical cutoff points were followed for mild (5), moderate (10), moderately severe (15), and severe (20) depression. The baseline data indicated good internal consistency (Cronbach α=.86).

The Kessler 10-item measure [42] was used to assess psychological distress at all time points. Cutoff points from Australian norms signify a severe risk of a clinical mental health condition for people who score >30, high risk for people who score between 22 and 29, moderate risk for people who score between 16 and 21, and low risk for people who score <15 [43]. The baseline data indicated good internal consistency (Cronbach α=.91).

The 15-item Mindful Attention and Awareness Scale [44] was used to measure the mindfulness of respondents at all time points. Mean responses across the 15 items were computed, with higher mean scores (range 1-6) indicating higher trait mindfulness. Internal consistency was good at baseline (Cronbach α=.91).

The 35-item, 8-dimension Assessment of Quality of Life (AQoL) measure [45], which assesses quality of life related to physical health (independent living, pain, and senses) and psychosocial health (mental health, happiness, coping, relationships, and self-worth), was used at all time points. Scores were computed using the 8-dimension AQoL algorithm (range 0.09-1.00). A score of 0.00 equates to death, and 1.00 equates to full health.

Perceptions of job demand, control, and support were used to assess work-related psychosocial risk at all time points. Demand and control were assessed using 7 items drawn from the Household, Income, and Labour Dynamics in Australia survey [46]. Scores were summed for 4 demand items (range 0-24) and 3 control items (range 0-18). A higher risk of job-related stress is indicated when demand scores are higher, and control scores are lower. Job support was assessed using summed responses to 6 items drawn from the Swedish Demand, Control, and Support Survey [47]. Higher scores (range 4-24) indicated a lower psychosocial risk of job stress. Internal inconsistency was weaker for the demand scale (Cronbach α=.65) than for the control (Cronbach α=.80) and support (Cronbach α=.80) measures.

Effects on productivity were based on estimates of health-related LPT [48]. Participants were asked to think about their work attendance in the previous 4 weeks and report the number of days they stayed away from work because of ill health (absentee days) and the number of days they went to work but were unwell (presenteeism days). Absentee days were considered 100% lost (eg, 2 absentee days=2 lost days). If presenteeism days were reported, an estimate of productivity (percentage) on those days was recorded. The number of lost productive days was assessed as the product of the number of presenteeism days and lost productivity on those days. For example, 3 presenteeism days at 60% productivity were calculated as follows:

(3 × [100−60]) = 1.2 lost days **(1)**

The total number of days lost through absenteeism and presenteeism is thus reported as health-related LPT.

The degree to which changes in participants’ mindful behaviors (eg, attentiveness, awareness, and acceptance) were noticeable to work colleagues was assessed at all time points using a 9-item observed mindfulness measure (OMM; [49]). This instrument includes items such as “The person has difficulty staying focused on what is happening to/around them as it occurs (Attentiveness),” “When asked how he or she is feeling, the person can identify their emotions easily (Awareness),” and “The person seems to recover well from unpleasant or stressful experiences (Acceptance).” Response options indicated the frequency of observed behaviors (1=not at all and 5=all the time). Scores for 3 items (items 1, 4, and 7) were reversed before summing to obtain subscale scores for observed mindful acceptance, awareness, and attentiveness and the total score. The internal consistency of OMM data at baseline was good (Cronbach α=.88).

A 16-item Organizational Citizenship Behaviors observer report instrument [50] was used at all time points to assess noticeable participant behaviors at work. Response options indicated the frequency of observed behaviors, and higher summed scores indicated higher degrees of altruism (range 5-30) and compliant behaviors (range 4-20). Cronbach test showed some internal inconsistency at baseline (altruism Cronbach α=.72 and compliance Cronbach α=.62).

Intervention adherence was assessed using self-reported seminar attendance and app use data from the Smiling Mind Workplace Program server. Whether the participants downloaded and engaged with the app (yes or no) was recorded. Engagement was calculated as the proportion of time spent in the Smiling Mind Workplace Program app activities out of a potential maximum of 343 minutes for the entire program. Participants’ perceptions of the acceptability of the intervention were assessed using qualitative data from 2 open questions in the T1 survey. Observers provided free-text responses at the end of each survey about their experience in the study and to share any additional information about their paired participants.

### Statistical Analysis

The required sample size was calculated using a pooled PSS estimate from a meta-analysis of 13 RCTs of workplace-based mindfulness programs (Cohen *d*=−0.54; mean difference −4.21, SE 0.14) [1]. A minimum of 198 participants was required to achieve a power of 0.8 and α=.025 (maintaining a family-wise error rate of 0.05) [51]. The recruitment target (n=261) allowed for 25% attrition.

Intention-to-treat analyses were conducted using an original assigned group approach [52]. Significance tests (α=.05) were adjusted using the Tukey method for multiple comparisons when >2 groups were included in the model. Analyses were conducted in the R (version 3.4.3; R Foundation for Statistical Computing) [53] using the psych [54], lme4 [55], and lmerTest packages [56]. Repeated measures linear mixed models were used to assess changes in the app+classes and app-only groups compared with the WLC group from T0 to T1, with age, sex, prior mindfulness training, and main occupation included to inform missing data computations. Two-group comparisons were used to test the difference in effect retention between the app+classes and app-only groups beyond T1. Cohen *d* standardized mean difference effect estimates were computed using the Lakens [57] guidelines (0.2=weak, 0.5=moderate, and 0.8=strong). Agreement between participants and their observers was assessed using intraclass correlation coefficient (ICC) estimates in 2-way random effects models following the Koo and Li [58] guidelines (0.5=poor, 0.5-0.75=moderate, 0.75-0.9=good, and >0.9=excellent agreement). Spearman correlations were used to test the relationship between program adherence and study outcomes. Chi-square and Fisher exact tests were used to explore the differences in intervention engagement and health-related LPT. Qualitative data were read twice by 2 authors (AJM and LB), with frequent themes identified, coded, and assessed using a content analysis approach [59].

## Results

### Participant Enrollment and Attrition

**Figure 1:**
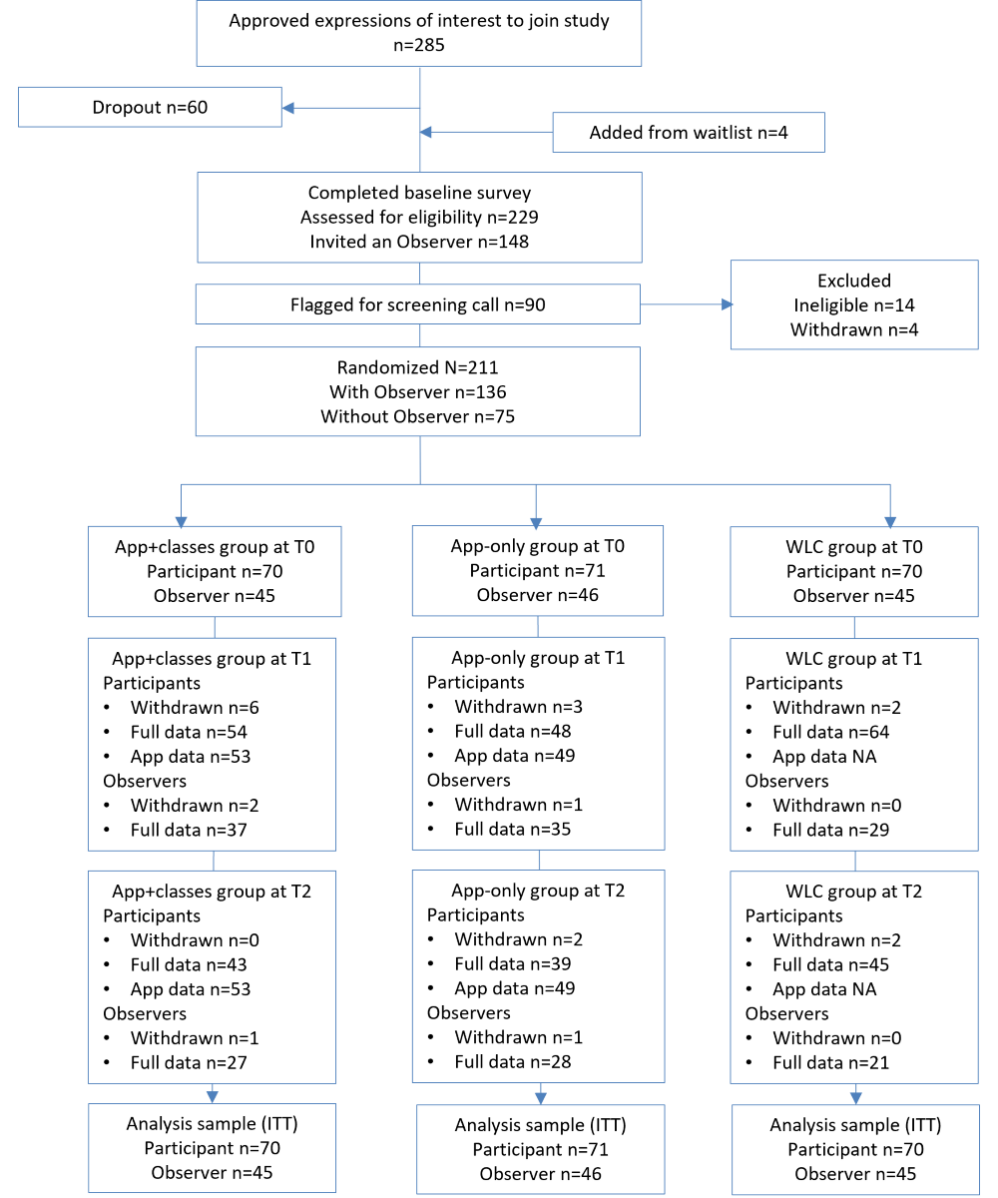
Participant flow diagram. Ineligible: did not meet inclusion criteria; Withdrawn: requested no further surveys, available data not withdrawn from analyses; ITT: intention to treat; T0: time point 0; T1: time point 1; T2: time point 2; WLC: waitlist control.

The flow of participants and observers is illustrated in Figure 1. Of an approved pool of 285 TSS employees, baseline measures were completed by 229 (80.4%) employees. Of the 229 respondents, 90 (39.3%) were invited to a screening interview by the study psychologist, of whom 14 (16%) were deemed clinically ineligible, an additional 4 (4%) withdrew, and 2 (2%) were excluded because of nonresponse. The starting sample of 211 individuals included 136 (64.5%) participants with paired observers. Group assignments were app+classes (participants 70/211, 33.2%; observers 45/136, 33.1%), app-only (participants 71/211, 33.6%; observers 46/136, 33.8%), and WLC (participants 70/211, 33.2%; observers 45/136, 33.1%). Statistical power for the hypothesized moderate-sized PSS effect was achieved in the starting sample.

Of the 211 participants and 136 observers, 15 (7.1%) participants and 6 (4.4%) observers advised withdrawal during the study period. The participants’ reasons for withdrawal were time pressures (4/15, 27%), changing job (4/15, 27%), difficulty accessing the app-based materials (1/15, 7%), extended leave (3/15, 20%), and no reason (3/15, 20%). Observers’ reasons included no longer being in contact with their paired participant (3/6, 50%) or their participant had withdrawn (3/6, 50%). Of the 211 participants, complete survey data were provided by 167 (79.1%) participants at T1 and 129 (61.1%) participants at T2.

### Participant Characteristics

Participant characteristics were similar across the intervention groups (Table 1), except for full-time workers. Just under half of the sample reported some prior exposure to mindfulness, and readiness to commence training was >80% across groups.

**Table 1:** Participant characteristics (n=211).

Characteristics variables			WLC^a^ (n=70)		App (n=71)		App+classes (n=70)		Difference (*P* value)^b^
**Age category (years), n (%)**									
	18 to 34	7 (10)		9 (13)		9 (13)		.60	
	35 to 44	18 (26)		20 (28)		23 (33)		.60	
	45 to 55	20 (29)		22 (31)		24 (34)		.60	
	55 to 64	23 (33)		17 (24)		14 (20)		.60	
	>65	2 (3)		3 (4)		0 (0)		.60	
Gender (female), n (%)			53 (76)		50 (70)		50 (71)		.76
**Educational attainment, n (%)**									
	High school	2 (3)		6 (9)		6 (9)		.37	
	College	24 (34)		16 (23)		19 (27)		.37	
	University	44 (63)		49 (69)		45 (64)		.37	
Living as married, n (%)			55 (79)		56 (79)		52 (74)		.77
Prior mindfulness training, n (%)			34 (49)		35 (49)		31 (44)		.81
**Main occupation, n (%)**									
	Blue collar	1 (1)		1 (1)		1 (1)		.21	
	Clerical or admin	15 (21)		5 (7)		12 (17)		.21	
	Technical or services	4 (6)		9 (13)		10 (14)		.21	
	Professional	38 (54)		48 (68)		35 (50)		.21	
	Senior manager	12 (17)		8 (11)		12 (17)		.21	
Works full time, n (%)			49 (70)		61 (86)		56 (80)		.07
**Work schedule, n (%)**									
	Regular daytime	64 (91)		61 (86)		62 (89)		.85	
	Regular evening or night	2 (3)		2 (3)		2 (3)		.85	
	Irregular or rotating	4 (6)		8 (11)		6 (9)		.85	
Percentage readiness for training, mean (SD)			86 (16)		85 (18)		82 (21)		.45

^a^WLC: waitlist control.

^b^Difference between-group *P* values computed using analysis of variance for continuous variables and chi-square tests of group equivalence for categorical variables.

### Aim 1: Intervention Effects for Perceived Stress

Postintervention RCT effect estimates are presented in Table 2. Although there was a downward trend in perceived stress, when compared with the WLC, there was no significant change for either the app+classes or app-only group. Prior exposure to mindfulness, readiness to commence training, or depression severity at baseline were not significant moderators.

**Table 2:** Postintervention randomized controlled trial effect estimates.

Outcome variables			Time point 0, mean (SE)^a^		Time point 1, mean (SE)		Effect estimates				
							β^a^ (SE)		*P* value^a,b^		Cohen *d*^c^ (95% CI)
**Perceived stress^d^**											
	WLC^e,f^	16.37 (0.75)		15.32 (0.77)		—^g^		—		—	
	App-only^h^	17.40 (0.74)		14.91 (0.84)		−1.44 (1.01)		.16		−0.06 (−0.39 to 0.27)	
	App+classes^i^	17.15 (0.75)		15.38 (0.81)		−0.73 (0.98)		.46		0.01 (−0.32 to 0.34)	
**Mindfulness^j^**											
	WLC	3.83 (0.09)		3.65 (0.10)		—		—		—	
	App-only	3.83 (0.09)		3.79 (0.10)		.15 (0.12)		.23		0.17 (−0.16 to 0.50)	
	App+classes	3.69 (0.09)		3.81 (0.10)		.31 (0.12)		.01		0.19 (−0.14 to 0.52)	
**Psychological distress^k^**											
	WLC	18.68 (0.67)		19.46 (0.68)		—		—		—	
	App-only	19.08 (0.66)		18.65 (0.73)		−1.21 (0.78)		.12		−0.14 (−0.47 to 0.19)	
	App+classes	19.21 (0.66)		18.22 (0.71)		−1.77 (0.75)		.02		−0.21 (−0.55 to 0.12)	
**Job demands**											
	WLC	16.41 (0.43)		15.64 (0.45)		—		—		—	
	App-only	16.79 (0.43)		15.90 (0.49)		−.13 (0.59)		.83		0.07 (−0.26 to 0.40)	
	App+classes	16.93 (0.43)		15.69 (0.47)		−.47 (0.57)		.41		0.01 (−0.32 to 0.34)	
**Job control**											
	WLC	10.11 (0.47)		10.45 (0.48)		—		—		—	
	App-only	10.67 (0.47)		11.25 (0.52)		.25 (0.55)		.65		0.19 (−0.14 to 0.52)	
	App+classes	10.60 (0.47)		11.03 (0.50)		.10 (0.53)		.86		0.14 (−0.19 to 0.47)	
**Job support**											
	WLC	18.43 (0.39)		18.40 (0.40)		—		—		—	
	App-only	17.85 (0.39)		18.70 (0.44)		.88 (0.50)		.08		0.09 (−0.24 to 0.42)	
	App+classes	18.03 (0.39)		18.08 (0.42)		.08 (0.48)		.87		−0.09 (−0.42 to 0.24)	
**QoL^l,m^: physical health**											
	WLC	0.75 (0.02)		0.75 (0.02)		—		—		—	
	App-only	0.76 (0.02)		0.77 (0.02)		.00 (0.02)		.83		0.12 (−0.21 to 0.45)	
	App+classes	0.75 (0.02)		0.76 (0.02)		.01 (0.02)		.74		0.06 (−0.27 to 0.39)	
**QoL: mental health**											
	WLC	0.37 (0.02)		0.39 (0.02)		—		—		—	
	App-only	0.37 (0.02)		0.43 (0.02)		.03 (0.02)		.13		0.24 (−0.09 to 0.57)	
	App+classes	0.35 (0.02)		0.40 (0.02)		.02 (0.02)		.26		0.06 (−0.27 to 0.39)	
**QoL: utility score**											
	WLC	0.71 (−0.02)		0.73 (0.02)		—		—		—	
	App-only	0.72 (−0.02)		0.76 (0.02)		.02 (0.02)		.28		0.18 (−0.15 to 0.51)	
	App+classes	0.69 (−0.02)		0.73 (0.02)		.02 (0.02)		.33		0.00 (−0.33 to 0.33)	

^a^Estimated marginal means and effect estimates from maximum likelihood linear mixed models with age, sex, education, and prior mindfulness exposure as auxiliary variables; all analyses were based on intention-to-treat principles with all cases analyzed in their original assigned group.

^b^Significant with α=.05.

^c^Standardized mean difference effect estimate computed using time point 1 estimated marginal means and SE.

^d^Perceived Stress Scale (10 items).

^e^WLC: waitlist control group.

^f^n=70.

^g^WLC ceased to be comparator after time point 1; hence, data are not shown.

^h^Self-guided app group (n=71).

^i^Self-guided app use plus supporting classes (n=70).

^j^Mindful Awareness and Attention Scale.

^k^Kessler-10 scale.

^l^QoL: quality of life.

^m^Assessment of Quality of Life (8 dimension).

**Figure 2:**
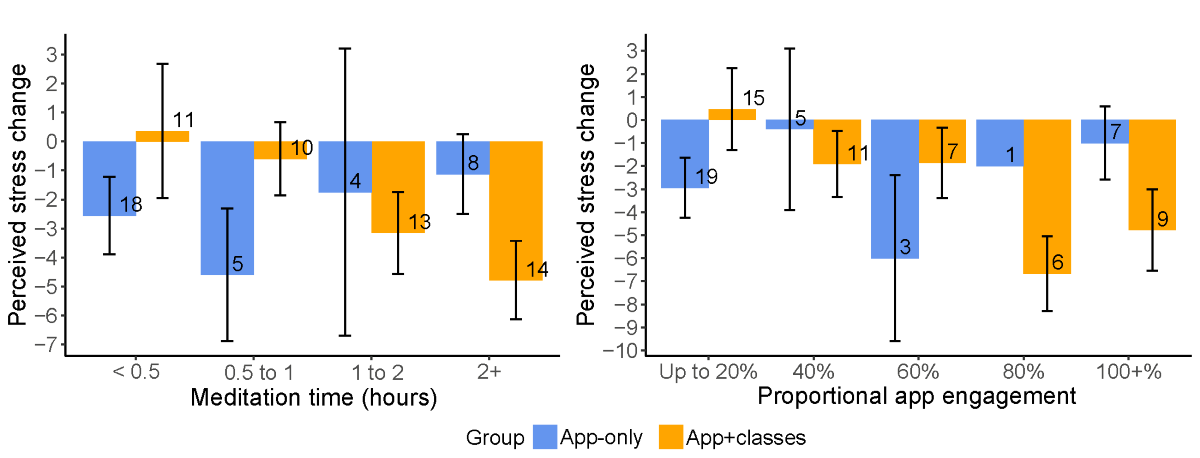
Perceived stress change from baseline to after the intervention by meditation time and app engagement.

Among the 70 participants in the app+classes group, class attendance diminished over time, with 45 (64%) attendees in the first class, 36 (51%) in the second, 33 (53%) in the third, and 32 (46%) in the fourth class. Table 3 shows that the Smiling Mind Workplace Program app was downloaded by 70% (49/70) of the participants in the app+classes group and 49% (35/71) of participants in the app-only group. The app+classes group also had higher median engagement with the learning and practice elements within the app (45/343 total activity minutes) and with the meditation practices over the 8-week period (73 meditation minutes) than those in the app-only group (45/343 total activity minutes, with 27 meditation minutes). Perceived stress change was significantly correlated with intervention engagement in the app+classes group (*r*=−0.33) but not in the app-only group. Investigation of T0:T1 change in PSS scores by meditation time and program engagement suggests an inverse linear dose–response pattern in the app+classes group. This pattern was not evident in the app-only group (Figure 2).

**Table 3:** Smiling Mind Workplace Program app engagement indices for the app+classes and app-only groups between time point 0 and time point 1^a^.

Engagement variables		App-only^b^ (n=71)	App+classes^c^ (n=70)	Test of difference (*P* value)
App downloads, n (%)		35 (49)	49 (70)	—^d^
**App use, median (IQR)**				
	Number lessons completed	2 (0-14)	4 (0-16)	.01
	Number activities completed	0 (0-4)	1 (0-7)	.09
	Total meditation minutes	27 (0-296)	73 (0-476)	.03
	Number meditations completed	4 (0-44)	11 (0-55)	.03
	Percentage of possible total engagement^e^	13% (0%-126%)	35% (1%-160%)	.05

^a^Tests of difference used 2-tailed *t* test using complete case data for normally distributed variables and Kruskal-Wallis rank sum test for nonnormally distributed variables.

^b^Self-guided app use.

^c^Self-guided app use plus classes.

^d^Not conducted.

^e^Total time if all app-based activities were completed was 343 minutes.

### Aim 2: Psychological Distress, Mindfulness, Work-Related Psychosocial Risks, and Quality of Life

The results (Table 2) show that compared with the WLC, the app+classes group reported small improvements in psychological distress (Cohen *d*=−0.21) and mindfulness (Cohen *d*=0.19). At T1, the Kessler-10 data showed that 15% (8/54) of respondents in the app+classes group transitioned into a lower category for risk of clinical mental health problems, whereas 2% (1/54) of participants shifted to a higher-risk category. No significant effects were found for either psychological distress or mindfulness in the app-only group, and an equal number reported beneficial (4/48, 8%) and detrimental changes in risk status (4/48, 8%). Of the 70 participants in the WLC, 14% (9/64) shifted to higher risk and 9% (6/64) to lower-risk categories during the initial intervention period.

No discernible trends in the quality of life data were evident for either the app+classes group or the app-only group when compared with the WLC group. Similarly, psychosocial risk factors did not change significantly in either active group at T1.

### Aim 3: Productivity and Workplace Incidents

The raw productivity and workplace incident results are presented in Table S1 of Multimedia Appendix 1. Health-related LPT was categorized into four levels: no health-related LPT, up to 1 day, 1 to 3 days, and >3 days. The app+classes and app-only groups trended lower in health-related LPT than in the WLC group following training, but the difference was not significant. The number of app+classes participants who reported work success increased from 26% (18/70) at T0 to 39% (17/43) at T2. This change was stronger than that observed in the app-only (28/71, 39% to 17/39, 43%) and WLC (18/70, 26% to 13/45, 29%) groups. Work failures reduced from T0 to T2 for the active groups (app+classes: 6/70, 9% to 3/43, 7%; app-only: 10/71, 14% to 4/39, 10%), whereas failures increased in the same period for the WLC (4/70, 6% to 4/45, 9%). Workplace accidents were infrequent in all groups, with 1% (1/70) of participants in the app+classes group, 7% (5/71) in the app-only group, and 6% (4/70) in the WLC group endorsing this item at T0.

### Aim 4: Observer-Reported Mindfulness and Organizational Citizenship

**Figure 3:**
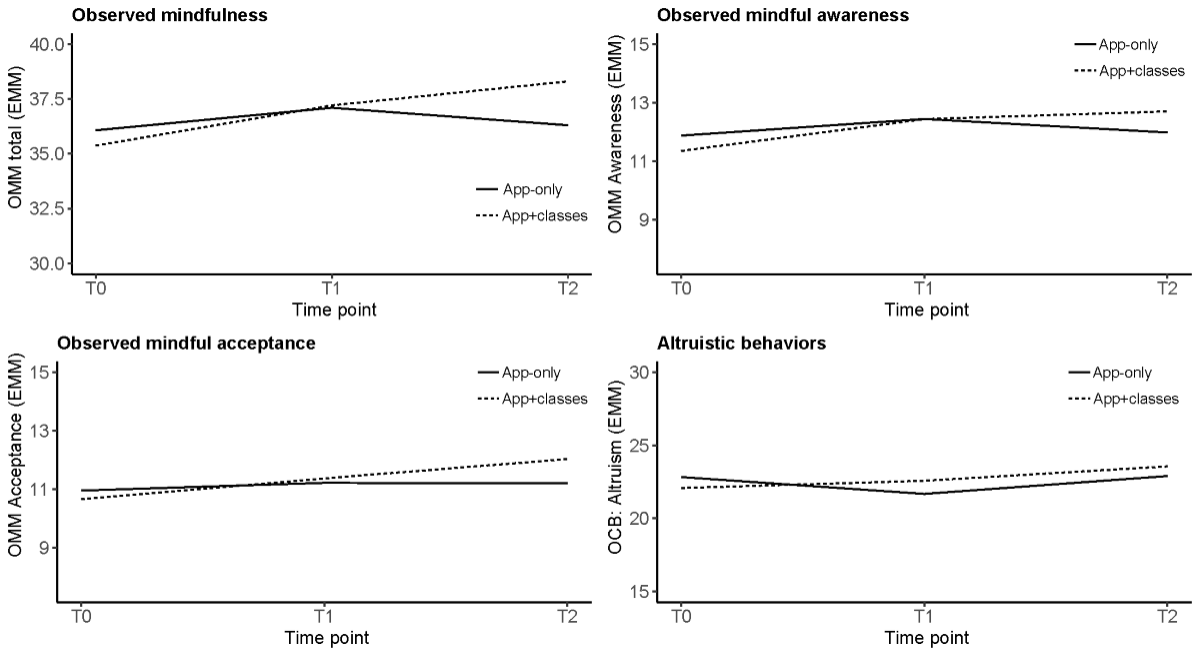
Change trends from baseline to 6 months: interactions between app-only and app+classes groups for observer-reported mindful and altruistic behaviours. Observed mindfulness measure (OMM) range 9 to 45; OMM awareness and acceptance range 3 to 15; and Organizational Citizenship Behavior Altruism subscale (OCB) range 5 to 30. EMM: estimated marginal mean; T0: time point 0; T1: time point 1; T2: time point 2.

Observer-reported outcomes are illustrated in Figure 3. The results are detailed in Table S2 of Multimedia Appendix 1. Changes in observer-reported mindful behaviors and self-reported mindfulness showed consistent agreement at each time point (T0: ICC=0.35, *P=*.01; T1: ICC=0.32, *P=*.03; T2: ICC=0.39, *P=*.03). At T1, observers reported a small but nonsignificant trend toward higher observed mindful behaviors in both active groups compared with the WLC. At the 6-month follow-up (T2), head-to-head comparison between the active groups showed that the app+classes participants displayed more noticeably mindful behaviors than the app-only participants (Cohen *d*=0.34, 95% CI −0.08 to 0.75).

The distribution of data in the organizational citizenship compliance subscale showed that responses were bounded at the top from baseline; thus, these data were excluded from the analyses. Although the results for altruism were not significant, plots (Figure 3) illustrate that the app+classes group trended higher on this measure at T1 and T2, whereas the app-only group initially trended toward lower altruism at T1, which was ameliorated at T2.

### Aim 5: Effect Retention

Results comparing the app+classes and app-only groups at the 6-month follow-up (T2) are reported in Table 4. The effects observed for mindfulness and psychological distress developed further in both groups beyond intervention completion (T1) such that there was no significant difference between groups at T2. The app+classes group continued to trend lower than the app-only group in perceived job demands and higher in job control from T1 to T2; however, the social support results observed at T1 showed no further development at T2.

**Table 4:** Effect estimates for the app+classes group compared with the app-only group at 6-months follow-up for mindfulness, psychological distress, job demands, and job control.

Outcome variable and group		T0^a^, mean^b^ (SE)^c^	T2^d^, mean (SE)	Effect estimate T0:T2	
				β^c^ (SE)	*P* value^c^
**Mindfulness**					
	App-only	3.82 (0.10)	3.91 (0.11)	Reference	Reference
	App+classes	3.68 (0.10)	3.94 (0.11)	.04 (0.16)	.82
**Psychological distress**					
	App-only	19.08 (0.70)	18.21 (0.79)	Reference	Reference
	App+classes	19.16 (0.70)	17.69 (0.78)	−.52 (1.11)	.64
**Job demands**					
	App-only	16.72 (0.44)	16.46 (0.52)	Reference	Reference
	App+classes	16.90 (0.44)	15.08 (0.51)	−1.38 (0.73)	.06
**Job control**					
	App-only	10.70 (0.45)	10.65 (0.53)	Reference	Reference
	App+classes	10.64 (0.46)	11.39 (0.52)	.73 (0.74)	.33

^a^T0: time point 1 (baseline).

^b^Estimated marginal means.

^c^β, SE, and *P* values from the 2-group comparison of effects in linear mixed models, with app-only group set as reference.

^d^T2: time point 2 (6-months from baseline).

### Intervention Acceptability

The frequency of themes derived from the qualitative data is reported in Table 5. Reports from the 2 active groups showed overall satisfaction with the mindfulness training. Responses to the free-text questions from the participants (57/141, 40.4%) indicated that they found the training useful, practical, helpful, or beneficial, more frequently among the app+classes (35/70, 50%) participants than app-only participants (22/71, 31%). Approximately 19% (13/70) of members of the app+classes group reported finding the program immediately beneficial, whereas this was volunteered by only 6% (4/71) of the app-only participants. The app was considered easy to use by 14.9% (21/141) of all participants. However, although 8.5% (12/141) of participants reported that they were incorporating the practice into daily life, 12.7% (18/141) of respondents found establishing a routine difficult, and 8.5% (12/141) of participants reported that it was not feasible to engage with the program while at work. Comments from 24% (17/70) of the app+classes group participants indicated that they found the seminars motivating. However, more app+classes group participants reported difficulties associated with time demands (5/70, 7%) and establishing a practice routine (12/70, 17%) than the app-only group participants (3/71, 4% and 6/71, 8%, respectively). A small number of participants reported technical problems with the app and seminars. One of the individuals in each group reported that they felt the research surveys were independently helpful in sensitizing them to their mental well-being. The in-app elements considered most useful by participants in both active groups were meditations, ranked highest by 57% (55/97) of respondents. Micropractices, which are brief mindful activities that can be used throughout the day, were rated very useful by 41% (40/97) of participants, in-app lessons by 32% (31/96) of participants, and body scan practices by 31% (30/97; data not shown).

**Table 5:** Frequency of themes derived from postintervention free-text responses regarding the usefulness of the program (N=141).

Themes derived from qualitative data			All respondents, n (%)		App+classes group (n=70), n (%)		App-only group (n=71), n (%)
**Participant view of outcomes**							
	Improved well-being	7 (5)		4 (6)		3 (4)	
	Improved sleep	4 (3)		2 (3)		2 (3)	
	Improved productivity	3 (2)		2 (3)		1 (1)	
	Improved recovery	2 (1)		1 (1)		1 (1)	
	Improved relationships	1 (1)		0 (0)		1 (1)	
**Acceptability**							
	Useful, practical, helpful, and beneficial	57 (40)		35 (50)		22 (31)	
	Immediate benefit and real-time application	17 (12)		13 (19)		4 (6)	
	Variety, choices, and range of app elements	11 (8)		7 (10)		4 (6)	
	Found app irritating and disruptive	6 (4)		2 (3)		4 (6)	
	Would recommend	4 (3)		3 (4)		1 (1)	
**Feasibility**							
	Easy to use, accessible, and flexible	21 (15)		9 (13)		12 (17)	
	Establishing routine is difficult	18 (13)		12 (17)		6 (8)	
	Seminars were motivating and beneficial	17 (12)		17 (24)		0 (0)	
	Incorporating practices into daily life	12 (9)		6 (9)		5 (7)	
	Not feasible at work	12 (9)		5 (7)		7 (10)	
	Technical problems with app	8 (6)		5 (7)		3 (4)	
	Time challenges or demands of training	8 (6)		5 (7)		3 (4)	
	Self-guided program difficult	7 (5)		1 (1)		6 (8)	
	Technical problems with seminars	3 (2)		3 (4)		0 (0)	
	No benefit from seminar attendance	3 (2)		3 (4)		0 (0)	
**Contextual circumstances**							
	Major life stresses during the study	10 (7)		5 (7)		2 (3)	
	Life got in the way (did not do training)	10 (7)		8 (11)		2 (3)	
	Did not use the app	8 (6)		0 (0)		8 (11)	
	Surveys made difference on their own	2 (1)		1 (1)		1 (1)	

### Data Availability Statement

The data that support the findings of this study are available on request from the corresponding author (LB).

## Discussion

### Principal Findings

This RCT assessed the effects of participating in a low-dose, app-based workplace-based mindfulness program delivered both with and without supporting classes in a sample of public sector employees. The study hypothesis that using the Smiling Mind Workplace Program app, either self-guided or with supporting classes, would result in moderate-sized reductions in perceived stress was not supported. Although the app+classes group engaged more with the training, neither group achieved the recommended dose. Despite the low engagement, when compared with the inactive control group, the app+classes group reported significant increases in mindfulness and decreases in psychological distress. These benefits were retained at 6-month follow-up, at which point the app+classes group also reported significantly lower perceived job demands than the app-only group. No significant effects were observed for either intervention group for health-related quality of life or productivity. Although the Smiling Mind Workplace Program app was well-received by most participants in the active groups, those whose training protocol was entirely self-guided engaged less with training and reported no statistically significant changes in any of the study outcomes.

The null result for perceived stress was unexpected, given consistent positive findings from other workplace-based mindfulness programs [1] and the apparent efficacy of the current intervention for significant and lasting benefits for psychological distress. Although the 2 constructs are usually correlated, they are not the same. Perceived stress refers to the perceived capacity to meet the demands of presenting stressors, whereas psychological distress refers to health risks associated with sustained or unrelieved stress [60]. It is plausible that participants in the app+classes group developed skills through their mindfulness training protocol to regulate their emotions, thereby attenuating distress, whereas their perception of the demands and frequency of stressors may have remained unchanged. The PSS results for all 3 groups, including the control, trended lower over the main intervention period (T0 to T1), which might suggest a sample-wide reduction in stressor exposure; however, this was not detected or reported in other data collected for this study.

The significant changes in mindfulness and distress were encouraging but lower than meta-analytic estimates from workplace-based mindfulness programs delivered via face-to-face classes or web-based learning platforms [61-63]. These findings support the likelihood of a dose–response relationship, where the degree of exposure to mindfulness training and practice is associated with the size of the effects [64]. Despite the lower effect sizes, the psychological distress scores at T1 suggest that the app+classes training protocol was sufficient to realize meaningful mental health risk reduction for 15% (8/54) of participants.

Higher engagement with the Smiling Mind Workplace Program app by app+classes participants appears to have been motivated by seminar attendance, a sentiment volunteered in free-text data by 24% (17/70) of app+classes participants. For example, one of the participants stated:

I was fortunate to be selected to attend sessions which I believe was VERY important. This helped tremendously with getting the motivation to work through the app sessions. Other colleagues from my work who were not selected to attend sessions have very low motivation and barely did any of the app sessions.

The self-guided app-only group not only missed the class-based educational and discursive opportunities but also engaged less than the app+classes participants with the in-app educational videos, lessons, and practice resources. This poorer engagement may explain the pattern in PSS changes depicted in Figure 2, where the app+classes group reported a clearer and more consistent dose–response than the app-only group. It is feasible that in the absence of feedback and guidance by a teacher, or the opportunity to discuss experiences with other learners, the app-only participants were less able to apply mindful awareness and acceptance, as their experiences arise and pass away during meditation practices, and thus derived less benefit [65].

The absence of significant improvement in mindfulness or distress in the app-only group indicates that self-guided use of the Smiling Mind Workplace Program app was insufficient to realize consistent changes within the main intervention period (T0 to T1). This finding is in keeping with previous work that has shown that face-to-face classes in the training protocol are associated with stronger improvements in mindfulness [64]. The continued development of mindfulness and reduction in psychological distress in the app-only group beyond T1 suggests that although classes boost training engagement and augment the benefits of app use, self-guided mindfulness training may still be beneficial with ongoing engagement; however, benefits may take longer to manifest.

Compared with the WLC group, no change was observed immediately after the intervention for either intervention group for participants’ perceptions of psychosocial risk factors, job demand, control, and support. However, at 6 months, the app+classes group reported a reduction in job demands that approached significance and a trend toward higher job control compared with the app-only group. Job demands and control are key factors associated with work-related stress in the theoretical job-demands-resources model, where it is the perception that demands outweigh available resources that leads to job strain. Job strain is understood to be responsible for a range of workplace health and performance problems [33]. Mindfulness training aims to cultivate adaptive coping skills and should thus be considered a secondary level strategy for workplace health and well-being [12]. However, in this study, it appears that higher mindfulness may also support changes in the way psychosocial stressors are perceived. Our findings for job demands (and the trends for job control) indicate that the Smiling Mind Workplace Program app, when supported with classes, might be protective against job strain by reducing perceptions of imbalance between work-related demands and improving personal resources and perceived control over work experiences [3]. The fact that these effects were evident only at the 6- month follow-up might mean that changed perceptions of work-related psychosocial risks emerge sequentially following the development of higher mindfulness.

An explanation for the sequential development of benefits following mindfulness training is provided in the Garland [66] Mindfulness to Meaning model. According to this model, the initial stages of learning mindfulness meditation can help reduce stress reactivity by developing attentional control; however, it is the sustained application of mindful awareness in meditation practice that cultivates acceptance and reappraisal skills. These skills, in turn, support regulatory and coping resources and are known to underpin positive affect and general well-being [5,15,67].

The null result for quality of life was unexpected, given that significant improvements were recorded on the briefer 4-dimension AQoL following the pilot face-to-face workplace-based mindfulness program in the same population [35]. Moreover, prior work has shown increased general well-being following workplace-based mindfulness programs [2], even when delivered via an app [27]. Findings from an RCT of the Wildflowers mindfulness app in a nonwork setting [32] reported that changes in mindful acceptance appear to take longer and require a greater amount of meditation practice than changes in stress and mood. It is feasible that the degree of engagement with the app+classes intervention in this study was sufficient for the acquisition of elementary mindfulness skills (attentional control and awareness) that support stress appraisals and that these changes underpinned the beneficial findings for distress and psychosocial risk factors (job demand and job control). However, the training dose appears to have been inadequate for developing skills associated with positive affect and general well-being, which are key factors associated with quality of life [66].

Trends in productivity data indicate that all 3 groups had decreased the number of health-related presenteeism and absenteeism days at the 6-month follow-up. Changes in productivity may also be sequential to changes in stress and mindfulness; however, our results did not show a causal link between mindfulness training and increased productivity. We propose that health-related LPT is an informative measure for assessing productivity effects in future workplace-based mindfulness program research, as higher mindfulness has been shown to alleviate psychological distress, depression, and anxiety, and these conditions are strongly associated with absenteeism and presenteeism [1,68].

The use of observer data to supplement self-reported changes in mindfulness and related behaviors addresses a limitation noted in approximately half of the published mindfulness studies [25]. Although the magnitude of interrater agreement was low, the consistent correspondence between self-reported mindfulness (Mindful Attention and Awareness Scale) and observer-reported mindful behaviors (OMM) strengthens the results reported in this study [69,70]. The work-based observers reported noticing increased mindful behaviors and a trend toward higher altruism among participants in the app+classes group but not in the app-only group at 6 months. These results lend weight to the potential for workplace-based mindfulness programs to have prosocial benefits in the workplace [18,71].

### Limitations, Strengths, and Future Research

There were timing and contextual considerations within our study. Baseline data collection coincided with the end of the summer break, a period during which many public sector employees are returning from annual leave. In contrast, the postintervention surveys coincided with political elections and major flooding in and around the state’s capital city, where many public sector employees are located. Thus, employee stress levels may have been lower than usual in the preintervention surveys and elevated after the intervention through these contextual factors.

The necessary lack of blinding and use of a waitlist rather than an active control means that nonspecific factors such as social desirability, expectancy, or experimenter effects cannot be ruled out as potential effect moderators. For example, our qualitative data appear to suggest that participants in the app-only group may have felt their lower dose training protocol to have a lower status than the app+classes protocol. Careful design of the WLC conditions in future research is recommended to help address this bias risk. Although an additional survey was conducted 14 months from baseline (time point 3), there was a very high degree of attrition, with only 15.2% (32/211) of the starting sample providing data. Follow-up analyses were therefore limited to the 6-month data. Raw data for productivity and workplace incidents are provided in Multimedia Appendix 1 to support future pooled analyses.

Strengths of this study include participant characteristics reflecting those of the broader TSS workforce, meaning the reported findings can be generalized to similar public sector workplaces with some confidence. Collecting objective app use data enabled us to overcome a reliance on self-report adherence to the training protocol; however, we did not record engagement with the Smiling Mind generic emails and were therefore not able to include exposure to this guiding material in our dose-exposed calculations. The use of observer reports was another strength of this study, although the ceiling effects in the organizational citizenship and observed mindfulness data prevented complete analyses. The use of multisource data increases confidence in self-reported study findings, and this study has shown that the collection and use of observer-reported data are both feasible and informative. We suggest that more studies collect observer reports to help build an evidence base around the effects of mindfulness training on workplace social and performance outcomes. More work is needed to understand the effects of mindfulness training on workplace productivity and health-related LPT.

### Conclusions

Despite the absence of effects for the primary study outcome, that is, perceived stress, the results for mindfulness, distress, and job demands support the Smiling Mind Workplace Program app as a workplace stress reduction intervention when supported by classes. Importantly, no evidence of adverse effects was observed from this low-dose mindfulness intervention. However, previous workplace mindfulness training research [1,2] indicates that workplace-based mindfulness programs with stronger engagement and higher training doses are likely to realize greater benefits, both for employees’ stress-related health and well-being and for organizational outcomes such as productivity and performance.

## Appendix

Multimedia Appendix 1 Supplementary materials.

Multimedia Appendix 2 CONSORT-eHEALTH checklist (V 1.6.1).

## Abbreviations

AQoL: Assessment of Quality of Life
CHERRIES: Checklist for Reporting Results of Internet e-Surveys
CONSORT: Consolidated Standards of Reporting Trials
ICC: intraclass correlation coefficient
LPT: lost productive time
OMM: observed mindfulness measure
PHQ-9: Patient Health Questionnaire-9
PSS: Perceived Stress Scale
RCT: randomized controlled trial
REDCap: Research Electronic Data Capture
T0: time point 0
T1: time point 1
T2: time point 2
TSS: Tasmanian State Service
TTC: Tasmanian Training Consortium
WLC: waitlist control
