# Supplementary material for: Effects of a Mindfulness App on Employee Stress in an Australian Public Sector Workforce: Randomized Controlled Trial
Source: JMIR Mhealth Uhealth. 2022 Feb 10;10(2):e30272. doi: 10.2196/30272 (PMC8874803; doi:10.2196/30272)
Supplement: Multimedia Appendix 1 [file mhealth_v10i2e30272_app1.pdf]

Bartlett, L. Martin, A., Kilpatrick, M., Otahal, P., Sanderson, K., Neil, A.L. (2022) Effects of a Mindfulness App on Employee Stress in an Australian Public Sector Workforce: Randomized Controlled Trial. JMIR mHealth and uHealth.

SUPPLEMENTARY ONLINE MATERIALS

Please find the full published article at: https://mhealth.jmir.org/2022/2/e30272

Recruitment materials: study invitation circulated by Tasmanian Training Consortium	pages 2-5
Smiling Mind Workplace Program - structure	pages 6-8
Supplementary Table S.1. Raw productivity and incident data by group and timepoint	page 9
Supplementary Table S.2. Intervention effect estimates by group and timepoint: observer-reported outcomes	page 10
Checklist for Reporting Results of Internet Surveys	page 11

RCT protocol: Appendix C.2.
Participant invitation

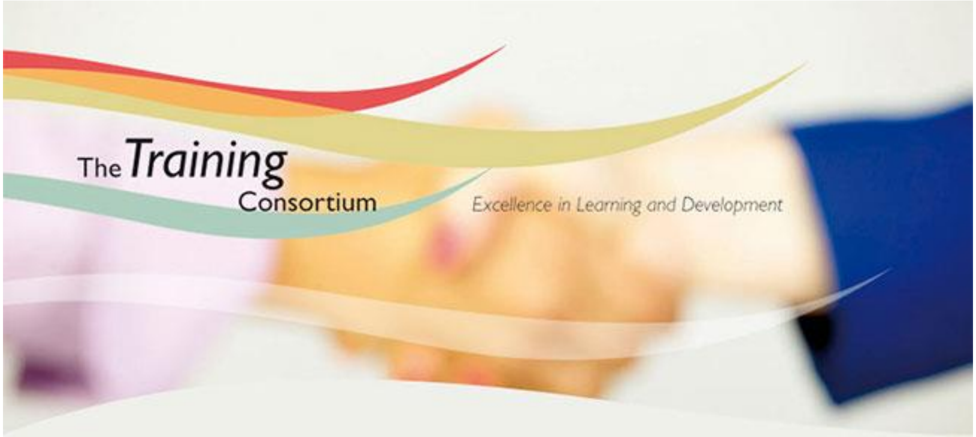

App-based Mindfulness Training

for employee stress protection

We know that work can be stressful, and that accumulated stress has poor health and wellbeing outcomes. We also know stress can lead to safety, conflict and productivity problems at work.

There is good evidence (including from a 2015 pilot study) that workplace-based mindfulness training can reduce stress and improve employee health and wellbeing. The pilot study results suggested that because of the wide range of work schedules and locations of the State Service workforce, flexible delivery might be more suitable than standard class-based training.

So, in the first half of 2018 the Smiling Mind Workplace App training program will be rolled out to about 400 public sector employees in Tasmania. The State Service Management Office, Department of Premier and Cabinet, has partnered with the University of Tasmania's Menzies Institute for Medical Research to study the effectiveness of this approach to stress management training.

Participating in the training requires that you register for

Program Details

Expression of Interest:

To register your interest for this training/study:

1. Review the **Study Information Pack** for participants and informants
2. Obtain approval to participate from your manager
3. **Register your interest**

Timeline:

30 November 2017 - registrations close

2018

the study, which involves completing four surveys over 12 months. Participants will be randomly assigned into 3 different training groups:

- Group A (App+ group) - self guided use of the App plus 4 x 1 hour seminars
- Group B (App group) - self guided use of the App without the need to attend seminars
- Group C (WLC group) - self guided use of the App plus 1 x 2 hour seminar. This group commences training after the other two groups have finished and will act as the study's wait-list control.

Once you have registered you will be asked to complete the baseline survey, which includes eligibility screening for the research. When you are accepted into the study you will be randomly allocated to a group and advised your training dates and details.

At the end of the study the survey data will be used to compare outcomes by group. All analyses are on de-identified data and no individual outcomes will be known or reported.

Participant requirements

- A smart phone, tablet or other device that can be used in personal time as well as at work
- A commitment to do the four surveys
- A commitment to attend seminars on the scheduled dates. Seminars for those in the App+ group will be held in Hobart and Launceston, with video conference attendance available in Burnie. Online participation for those located in more remote areas may be arranged.

You will be invited to nominate up to two work-based people (informants), who are willing to respond to a series of brief survey questions about you four times over the 12-month study period. Nominating informants is not compulsory, but it is encouraged as it will help us understand any social effects that arise following training.

16 February - survey 1 completed

23 February - groups allocated

2 March - App available to App and App+ groups

6 March - seminar 1 for App+ group

20 March - seminar 2 for App+ group

27 March - seminar 3 for App+ group

10 April - seminar 4 for App+ group

4 May - survey 2 completed

15 May - seminar and App available to WLC group

18 August - survey 3 completed

22 February 2019 - survey 4 completed

Print this flyer

Cost

$95.00 (GST inc) this covers administration and delivery of the seminars and a 12 month licence for the App. This fee will be covered by

About the App

All participants will receive a 12 month licence to the *Smiling Mind* workplace App. This App is different to *Smiling Mind's* freely available one. It is richer in content, uses explanatory videos and is designed for use by working adults. The App has been designed by organisational psychologists to address challenges common in today's working environments.

Module 1 - Everyday Mindfulness (Introduction, moving with awareness)
Module 2 - Calm (Focusing on stress, transition and change management)
Module 3 - Clarity (Building concentration and focus)
Module 4 - Connection (With yourself, others and bringing out the inner leader)
Module 5 - Mindful Mastery (Consolidating skills and building routine)

Features of the App include:

- Exercises that develop skills for detecting and coping with stress
- Activities aimed at cultivating concentration and focus, managing change and transition, and building leadership attributes
- Daily practices such as brain break and sitting exercises, with about 30 brief guided mindfulness meditations customised for use at work
- Practical activities to help bring moments of informal mindfulness into the everyday, such as moving with awareness between meetings, breathing techniques, listening exercises
- Regular emails with tips from an experienced mindfulness teacher to help embed learning into daily life.

your employer

Terms and Conditions

Expressions of interest are subject to agency review and approval of funding.

Acceptance into the study is subject to a suitability screening process undertaken after completing the first survey.

More information:

P 03 6232 7511
E ttc@dpac.tas.gov.au

To view all courses visit www.ttc.tas.gov.au

You are directed to information on how your personal information is protected.
See also the disclaimer and copyright notice governing the information provided.

Tasmania
Explore the possibilities

Bartlett, L. Martin, A., Kilpatrick, M., Otahal, P., Sanderson, K., Neil, A.L. (2022) Effects of a Mindfulness App on Employee Stress in an Australian Public Sector Workforce: Randomized Controlled Trial. JMIR mHealth and uHealth.
SUPPLEMENTARY ONLINE MATERIALS

The Smiling Mind Workplace Program – history, structure and contents

The archive, source code and algorithms for the SMWP app are the commercial property of Smiling Mind. Please contact Smiling Mind directly to gain access for review - info@smilingmind.com.

Smiling Mind is a not-for-profit Australian organisation founded in 2012. The marketed SMWP, which utilises the SMWP App, was designed by organisational psychologists from IBM Australia New Zealand and mindfulness expert and psychologist Olivia Downing. The objective of the SMWP is to enable working adults to develop mindfulness skills and embed mindfulness practices into daily life. The program was developed using an iterative approach incorporating data obtained through employee interviews, organisational review and extensive pilot testing. An internal (unpublished) evaluation of participant satisfaction with the program, user experience and pre-post efficacy for stress, wellbeing and productivity preceded release of the market-based SMWP in 2014.

The established SMWP runs for eight weeks and involves a series of learning modules delivered in four interactive one-hour workshops led by a Smiling Mind facilitator. Each workshop has a key message: choosing to respond mindfully to stressors; remaining calm in the face of demands; managing attention wisely; and cultivating good relationships with the self and others. The workshops are run in conjunction with self-guided use of the SMWP App. The SMWP App includes 41 elements containing the videos and audio lessons, guided meditations and practical activities such as moving with awareness between meetings, breathing techniques and listening exercises to help cultivate workplace mindfulness. Use of the SMWP App is supported by an eight-week communications strategy, which comprises fortnightly generic emails relating to the content covered in the workshops and app-based lessons. The recommended minimum engagement with the SMWP App includes undertaking between 10 and 20 minutes per day of guided mindfulness meditation practice and/or activities.

The Smiling Mind Workplace Program: App contents

Module	Content and practices
Introduction to Mindfulness	What is Mindfulness?<br>Get started: 1 Minute exercise<br>The next step: 5 Minute exercise
Module 1 – Everyday Mindfulness	VIDEO – Everyday Mindfulness<br>Brain break: the breath<br>Daily 7 Minute Sitting Practice<br>Moving with Awareness<br>Daily Body Scan<br>Eating with Awareness<br>Practical activity 1: S.T.O.P and breathe
Module 2 - Calm	VIDEO - CALM<br>Brain break: Pause & Reset<br>Stress Management<br>Managing Transitions and Change<br>Work wind down<br>Sleep: rest, relax, dream.<br>Practical activity 2: Meditation Corridor/Stairs

Module 3 - Clarity	VIDEO - CLARITY<br>Brain break: Take a closer look<br>Starting your day<br>Daily 15 Minute Sitting Practice<br>Concentration and Focus<br>Creativity<br>Practical activity 3: Curiosity
Module 4 - Connection	VIDEO - CONNECTION<br>Daily Brain Break<br>Daily 7 Minute Sitting Practice<br>Connecting with Yourself<br>Connecting with Others<br>Connecting with your Inner Leader<br>Practical activity 4: Deep Listening
Module 5 - Mindfulness Mastery	Building resilience<br>Decision making<br>Befriending your inner critic<br>Communicating mindfully
Daily Practices	3 Minute Brain Break<br>7 Minute Sitting Practice<br>15 Minute Sitting Practice<br>15 Minute Body Scan

The Smiling Mind Workplace App+ Seminar Series

Workshop 1: Introduction

The key message is that we as individuals can choose how we respond to the stressors we find at work and at home and can manage our modern lives with more Clarity, Calm and Connection through bringing the skill of mindfulness into our lives.

The introductory session will address the following areas:

- Why is mindfulness and meditation important in business now?
- Impacts of stress on health, happiness and productivity
- A brief overview of the science of mindfulness
- Key benefit of mindfulness at work
- What is mindfulness and what is meditation?
- How do we practice mindfulness formally and informally at home and at work?
- Default vs. Active mode of the brain
- Formal meditation practice and debrief
- 6 Key Mindfulness Mindsets
- Neuroplasticity and mindfulness and the brain
- Practical Mindfulness Tool Kit
- Individual Commitments to bringing mindfulness into the work day

Workshop 2: Calm

The key message in this session is that even though there are more demands placed on us now than any other time in history due to, among other things, technology, globalization and constant change we can manage our stress and emotional responses with more ease through practicing mindfulness.

- Reflection of personal responses to stress
- Overview of flight or fight response
- Stress and Performance – how can stress serve us?
- Emotions and Emotional Intelligence at work
- Mindfulness Mindsets – Non-judgement and Letting Go
- Formal Mindfulness Practice
- Practical Tools – Mindsets, Practices and Actions
- Individual commitments to bringing more Calm into the workplace

Workshop 3: Clarity

The key message in this session is that we all have access to more mental horsepower than we are currently using, we just need to learn how to manage our attention more wisely. Mindfulness and meditation help us to do this.

- What is the impact of mindlessness on our performance?
- Exploring when individuals feel most in flow
- Mindfulness can upgrade the hardware of the brain
- Focus and Memory
- Creativity and Innovation
- Mindfulness Mindsets – Beginners' Mind & Non-Striving
- Formal Meditation Practice
- Intuitive decision making and problem solving
- Practical Tools – Mindsets, Practices and Actions
- Individual Commitments to bringing more Clarity into the workplace

Workshop 4: Connection

The key message for this session is that we are not currently maximizing the access we could have to deeper relationships with both ourselves and others. Building our Mindfulness skills can support us to develop more self-awareness and self-respect and in turn more collaborative team relationships as well as authentic leadership skills.

- We are all wired for connection – mindfulness can help us to satisfy this need
- Formal Meditation Practice
- Building self-awareness (the foundation of emotional intelligence) and self-respect
- Mindfulness Mindsets – Compassion & Acceptance
- Teamwork and Mindful Listening
- How to become and authentic leader
- Practical Tools – Mindsets, Practices and Actions

- Individual Commitments to bringing more Connection into the workplace
- 4-Session Program Review
- Organizational Mindfulness Initiatives

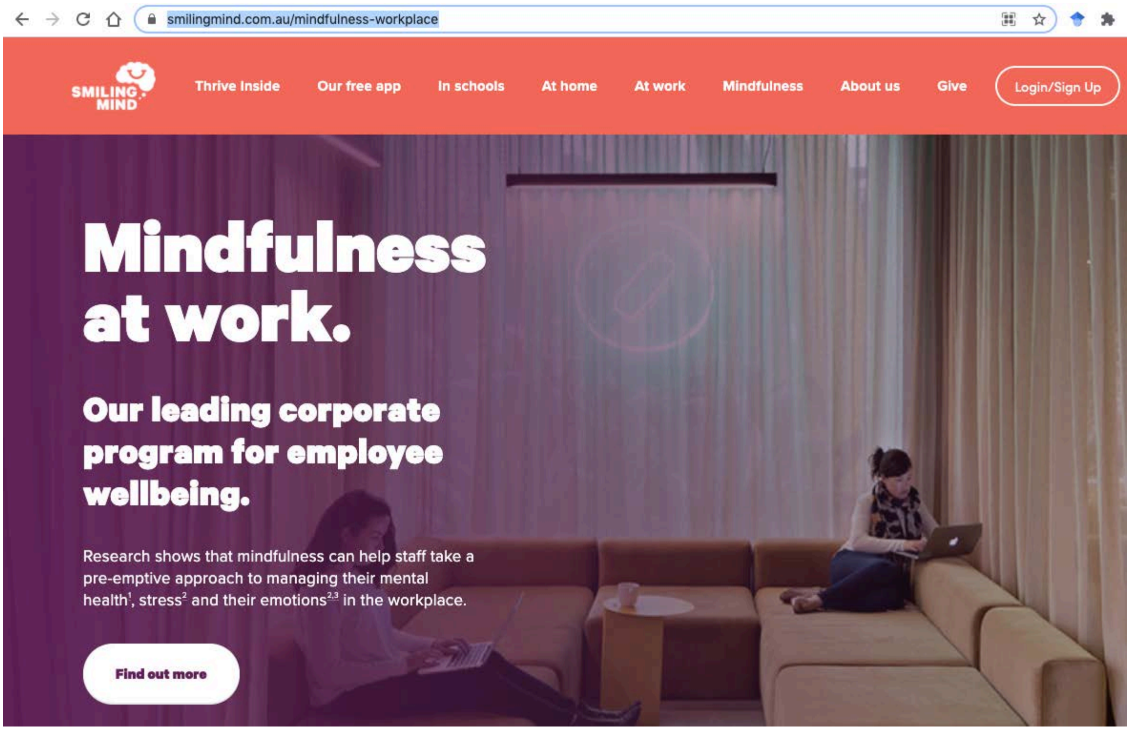

Supplementary Table S.1. Raw productivity and workplace incident data by group and timepoint

Group	WLC						App						App+					
Timepoint (n responses)	T0 (n=70)		T1 (n=64)		T2 (n=45)		T0 (n=71)		T1 (n=48)		T2 (n=39)		T0 (n=70)		T1 (n=55)		T2 (n=43)	
Health-related lost productive days, n (%)																		
None	44	(63)	30	(43)	36	(51)	36	(51)	39	(55)	47	(66)	39	(56)	43	(61)	49	(70)
Up to one day	11	(16)	18	(26)	13	(19)	16	(23)	10	(14)	3	(4)	12	(17)	10	(14)	4	(6)
One to three days	10	(14)	15	(21)	7	(10)	12	(17)	13	(18)	15	(21)	13	(19)	9	(13)	10	(14)
More than three days	5	(7)	7	(10)	14	(20)	7	(10)	9	(13)	6	(9)	6	(9)	8	(11)	7	(10)
Absenteeism days, n (%)																		
None	56	(80)	40	(63)	30	(60)	54	(76)	36	(69)	27	(66)	54	(77)	40	(70)	30	(67)
Up to two days	13	(19)	19	(30)	11	(22)	12	(17)	11	(21)	11	(27)	13	(19)	11	(19)	10	(22)
Two to five days	1	(1)	3	(5)	5	(10)	3	(4)	3	(6)	2	(5)	3	(4)	5	(9)	4	(9)
More than five days	0	0	2	(3)	4	(8)	2	(3)	2	(4)	1	(2)	0	0	1	(2)	1	(2)
Presenteeism days, n (%)																		
None	41	(59)	32	(50)	21	(42)	41	(58)	22	(42)	20	(49)	43	(61)	32	(56)	28	(62)
Up to two days	16	(23)	12	(19)	10	(20)	14	(20)	11	(21)	8	(20)	12	(17)	13	(23)	12	(27)
Two to five days	8	(11)	16	(25)	15	(30)	11	(16)	12	(23)	8	(20)	8	(11)	6	(11)	3	(7)
More than five days	5	(7)	4	(6)	4	(8)	5	(7)	7	(14)	5	(12)	7	(10)	6	(11)	2	(4)
Productivity on presenteeism days, n (%)																		
100% productive	47	(76)	33	(60)	22	(51)	41	(63)	23	(54)	20	(54)	44	(70)	33	(73)	28	(76)
75% or more	6	(10)	13	(24)	8	(19)	9	(14)	10	(23)	11	(30)	13	(21)	6	(13)	4	(11)
50% to 75%	7	(11)	9	(16)	11	(26)	15	(23)	10	(23)	5	(14)	6	(10)	5	(11)	4	(11)
50% or less	2	(3)	0	0	2	(5)	0	0	0	0	1	(3)	0	0	1	(2)	1	(3)
Work successes, n (%)	18	(26)	19	(30)	13	(28)	28	(39)	18	(38)	17	(42)	18	(26)	21	(38)	17	(39)
Work failures, n (%)	4	(6)	7	(11)	4	(9)	10	(14)	8	(17)	4	(10)	6	(9)	2	(4)	3	(7)
% Impacted, M (SD)	71	(9)	78	(9)	75	(8)	73	(21)	61	(26)	82	(11)	65	(14)	81	(1)	78	(20)
% Recovered, M (SD)	60	(30)	41	(13)	76	(31)	51	(25)	63	(40)	41	(28)	70	(22)	87	(18)	66	(46)
Work accidents, n (%)	4	(6)	3	(5)	2	(4)	5	(7)	3	(6)	2	(5)	1	(1)	0	-	1	(2)
% Impacted, M (SD)	57	(42)	62	(16)	63	(18)	85	(14)	83	(9)	74	(3)	90	-	-	-	34	-
% Recovered, M (SD)	74	(29)	69	(9)	98	(4)	80	(20)	34	(49)	78	(4)	25	-	-	-	87	-

WLC: Wait-list control group; App: Self-guided App use; App+: Self-guided App use supported with classes; T0: Baseline; T1: Post-intervention; T2: Six-months from baseline

Table S.2. Intervention effect estimates by group and timepoint: observer-reported outcomes

Outcomes	Estimated marginal means by timepoint						T0:T1 Effect estimates					T0:T2 Effect estimates				
	T0		T1		T2		App+ and App vs WLC					App+ vs App				
OMM total	M	SE	M	SE	M	SE	β	SE	p	d	95%CI	β	SE	p	d	95% CI
WLC	35.31	(0.86)	36.05	(0.91)			REF									
App	36.08	(0.82)	37.09	(0.84)	36.31	(0.89)	0.27	(0.88)	0.77	0.18	-0.23, 0.59	REF				
App+	35.38	(0.82)	37.21	(0.83)	38.31	(0.89)	1.09	(0.87)	0.22	0.20	-0.21, 0.62	2.71	(0.97)	0.01*	0.34	-0.08, 0.75
OMM Awareness																
WLC	11.50	(0.36)	11.73	(0.38)			REF									
App	11.88	(0.34)	12.43	(0.35)	11.98	(0.37)	0.33	(0.43)	0.44	0.29	-0.13, 0.70	REF				
App+	11.35	(0.34)	12.44	(0.35)	12.70	(0.37)	0.86	(0.42)	0.04	0.29	-0.12, 0.71	1.25	(0.44)	0.01*	0.29	-0.12, 0.70
OMM Acceptance																
WLC	10.56	(0.37)	10.82	(0.40)			REF									
App	10.95	(0.35)	11.22	(0.37)	11.20	(0.38)	0.01	(0.45)	0.98	0.16	-0.26, 0.57	REF				
App+	10.65	(0.35)	11.37	(0.36)	12.03	(0.38)	0.46	(0.45)	0.31	0.22	-0.20, 0.63	1.13	(0.43)	0.01*	0.33	-0.09, 0.74
OCB Altruism																
WLC	22.25	(0.79)	21.27	(0.85)			REF									
App	22.83	(0.75)	21.61	(0.78)	22.89	(0.83)	-0.24	(0.97)	0.81	0.06	-0.35, 0.47	REF				
App+	22.08	(0.75)	22.57	(0.78)	23.56	(0.84)	1.47	(0.96)	0.13	0.24	-0.17, 0.65	1.41	(1.03)	0.17	0.12	-0.29, 0.53

Mean, SE, β, p: Estimated marginal means and effect estimates from maximum likelihood linear mixed models with age, sex, education and prior mindfulness exposure as auxiliary variables; * significant with α=0.05; d: Cohen's Standardized Mean Difference effect estimate computed using EMMEANS and SE. OMM: Observed Mindfulness Measure, range 9-45); OMM Awareness and Acceptance dimensions, range 3-15; OCB Altruism: Organisational Citizenship Behaviours Altruism dimension, range 5-30. WLC: Wait-List Control; App+ and App: active intervention group

Bartlett, L. Martin, A., Kilpatrick, M., Otahal, P., Sanderson, K., Neil, A.L. (2022) Effects of a Mindfulness App on Employee Stress in an Australian Public Sector Workforce: Randomized Controlled Trial. JMIR mHealth and uHealth. https://mhealth.jmir.org/2022/2/e30272

SUPPLEMENTARY ONLINE MATERIALS

Checklist for Reporting Results of Internet E-Surveys (CHERRIES)

Checklist Item	***Explanation***	***Page Number***
Describe survey design	Describe target population, sample frame. Is the sample a convenience sample? (In "open" surveys this is most likely.)	p.4
IRB approval	Mention whether the study has been approved by an IRB.	p.4
Informed consent	Describe the informed consent process. Where were the participants told the length of time of the survey, which data were stored and where and for how long, who the investigator was, and the purpose of the study?	p.4-5
Data protection	If any personal information was collected or stored, describe what mechanisms were used to protect unauthorized access.	p.4 and supplementary
Development and testing	State how the survey was developed, including whether the usability and technical functionality of the electronic questionnaire had been tested before fielding the questionnaire.	p.5
Open survey versus closed survey	An "open survey" is a survey open for each visitor of a site, while a closed survey is only open to a sample which the investigator knows (password-protected survey).	Surveys were closed.
Contact mode	Indicate whether or not the initial contact with the potential participants was made on the Internet. (Investigators may also send out questionnaires by mail and allow for Web-based data entry.)	No
Advertising the survey	How/where was the survey announced or advertised? Some examples are offline media (newspapers), or online (mailing lists – If yes, which ones?) or banner ads (Where were these banner ads posted and what did they look like?). It is important to know the wording of the announcement as it will heavily influence who chooses to participate. Ideally the survey announcement should be published as an appendix.	p.4
Web/E-mail	State the type of e-survey (eg, one posted on a Web site, or one sent out through e-mail). If it is an e-mail survey, were the responses entered manually into a database, or was there an automatic method for capturing responses?	p.4-5
Context	Describe the Web site (for mailing list/newsgroup) in which the survey was posted. What is the Web site about, who is visiting it, what are visitors normally looking for? Discuss to what degree the content of the Web site could pre-select the sample or influence the results. For example, a survey about vaccination on a anti-immunization Web site will have different results from a Web survey conducted on a government Web site	N/A
Mandatory/voluntary	Was it a mandatory survey to be filled in by every visitor who wanted to enter the Web site, or was it a voluntary survey?	voluntary

Incentives	Were any incentives offered (eg, monetary, prizes, or non-monetary incentives such as an offer to provide the survey results)?	No
Time/Date	In what timeframe were the data collected?	p.4
Randomization of items or questionnaires	To prevent biases items can be randomized or alternated.	N/A
Adaptive questioning	Use adaptive questioning (certain items, or only conditionally displayed based on responses to other items) to reduce number and complexity of the questions.	Yes
Number of Items	What was the number of questionnaire items per page? The number of items is an important factor for the completion rate.	Each questionnaire had a separate survey page.
Number of screens (pages)	Over how many pages was the questionnaire distributed? The number of items is an important factor for the completion rate.	A maximum of 12 pages of questions was presented.
Completeness check	It is technically possible to do consistency or completeness checks before the questionnaire is submitted. Was this done, and if "yes", how (usually JAVAScript)? An alternative is to check for completeness after the questionnaire has been submitted (and highlight mandatory items). If this has been done, it should be reported. All items should provide a non-response option such as "not applicable" or "rather not say", and selection of one response option should be enforced.	Yes, completeness was assessed at data analysis
Review step	State whether respondents were able to review and change their answers (eg, through a Back button or a Review step which displays a summary of the responses and asks the respondents if they are correct).	Participants could go back to view responses prior to submitting the final survey.
Unique site visitor	If you provide view rates or participation rates, you need to define how you determined a unique visitor. There are different techniques available, based on IP addresses or cookies or both.	Unique codes applied by RedCap linked the email addresses to the participants' study identifier and

		ensured survey access was per protocol.
View rate (Ratio of unique survey visitors/unique site visitors)	Requires counting unique visitors to the first page of the survey, divided by the number of unique site visitors (not page views!). It is not unusual to have view rates of less than 0.1 % if the survey is voluntary.	N/A
Participation rate (Ratio of unique visitors who agreed to participate/unique first survey page visitors)	Count the unique number of people who filled in the first survey page (or agreed to participate, for example by checking a checkbox), divided by visitors who visit the first page of the survey (or the informed consents page, if present). This can also be called "recruitment" rate.	N/A
Completion rate (Ratio of users who finished the survey/users who agreed to participate)	The number of people submitting the last questionnaire page, divided by the number of people who agreed to participate (or submitted the first survey page). This is only relevant if there is a separate "informed consent" page or if the survey goes over several pages. This is a measure for attrition. Note that "completion" can involve leaving questionnaire items blank. This is not a measure for how completely questionnaires were filled in. (If you need a measure for this, use the word "completeness rate".)	Attrition was tracked throughout the study because the sample was known.
Cookies used	Indicate whether cookies were used to assign a unique user identifier to each client computer. If so, mention the page on which the cookie was set and read, and how long the cookie was valid. Were duplicate entries avoided by preventing users access to the survey twice; or were duplicate database entries having the same user ID eliminated before analysis? In the latter case, which entries were kept for analysis (eg, the first entry or the most recent)?	No
IP check	Indicate whether the IP address of the client computer was used to identify potential duplicate entries from the same user. If so, mention the period of time for which no two entries from the same IP address were allowed (eg, 24 hours). Were duplicate entries avoided by preventing users with the same IP address access to the survey twice; or were duplicate database entries having the same IP address within a given period of time eliminated before analysis? If the latter, which entries were kept for analysis (eg, the first entry or the most recent)?	No

Log file analysis	Indicate whether other techniques to analyze the log file for identification of multiple entries were used. If so, please describe.	No
Registration	In “closed” (non-open) surveys, users need to login first and it is easier to prevent duplicate entries from the same user. Describe how this was done. For example, was the survey never displayed a second time once the user had filled it in, or was the username stored together with the survey results and later eliminated? If the latter, which entries were kept for analysis (eg, the first entry or the most recent)?	Surveys were only available if the respondent used the link embedded in the invitation email and were unavailable after completion.
Handling of incomplete questionnaires	Were only completed questionnaires analyzed? Were questionnaires which terminated early (where, for example, users did not go through all questionnaire pages) also analyzed?	No, all data were included in analyses.
Questionnaires submitted with an atypical timestamp	Some investigators may measure the time people needed to fill in a questionnaire and exclude questionnaires that were submitted too soon. Specify the timeframe that was used as a cut-off point, and describe how this point was determined.	N/A
Statistical correction	Indicate whether any methods such as weighting of items or propensity scores have been used to adjust for the non-representative sample; if so, please describe the methods.	No

This checklist has been modified from Eysenbach G. Improving the quality of Web surveys: the Checklist for Reporting Results of Internet E-Surveys (CHERRIES). J Med Internet Res. 2004 Sep 29;6(3):e34 [erratum in J Med Internet Res. 2012; 14(1): e8.]. Article available at https://www.jmir.org/2004/3/e34/; erratum available https://www.jmir.org/2012/1/e8/. Copyright ©Gunther Eysenbach. Originally published in the Journal of Medical Internet Research, 29.9.2004 and 04.01.2012.

This is an open-access article distributed under the terms of the Creative Commons Attribution License (https://creativecommons.org/licenses/by/2.0/), which permits unrestricted use, distribution, and reproduction in any medium, provided the original work, first published in the Journal of Medical Internet Research, is properly cited.
